# Chemical composition and antimicrobial activity of essential oils obtained from leaves and flowers of *Salvia hydrangea* DC. ex Benth.

**DOI:** 10.1038/s41598-020-73193-y

**Published:** 2020-09-24

**Authors:** Mansureh Ghavam, Maria Letizia Manca, Maria Manconi, Gianluigi Bacchetta

**Affiliations:** 1 Department of Range and Watershed Management, Faculty of Natural Resources and Earth Sciences, University of Kashan, Kashan, Iran; 2 Department Life and Environmental Sciences, University of Cagliari, Cagliari, Italy; 3 Hortus Botanicus Karalitanus (HBK), University of Cagliari, Cagliari, Italy

**Keywords:** Environmental biotechnology, Bioinorganic chemistry

## Abstract

The majority of essential oils obtained from vascular plants have been demonstrated to be effective in treating fungal and bacterial infections. Among others, *Salvia hydrangea* is an endemic half-shrub belonging to the Lamiaceae family that has been widely used from ancient times in Iranian traditional medicine. The aim of this study was to compare the composition and antimicrobial properties of essential oils obtained from leaves or flowers of this plant, collected from the Daran region of Iran during June 2018. The oils were obtained using Clevenger apparatus, their composition was evaluated by means of gas chromatography/mass spectrometry (GC/MS) and the antimicrobial properties were assayed by measuring inhibition halos, minimum inhibitory concentration (MIC) and minimum bactericidal concentration (MBC). The yield of leaf oil was ~ 0.25% and that of flower oil was ~ 0.28%. Oil composition was affected by the part of the plants used: the most abundant bioactives contained in leaf essential oil were (+)-spathulenol (16.07%), 1,8-cineole (13.96%), trans-caryophyllene (9.58%), β-pinene (8.91%) and β-eudesmol (5.33%) and those in flower essential oil were caryophyllene oxide (35.47%), 1,8-cineole (9.54%), trans-caryophyllene (6.36%), β-eudesmol (4.11%), caryophyllenol-II (3.46%) and camphor (3.33%). Both the oils showed a significant inhibitory and lethal effect on the Gram-negative bacteria *Pseudomonas aeruginosa* (MIC ~ 16 µg/mL), *Shigella dysenteriae* and *Klebsiella pneumoniae* (MIC ~ 62 µg/mL). Therefore, the essential oils obtained from both leaves and flowers of *S. hydrangea* may have potential application as bactericidal agents against some bacteria.

## Introduction

Essential oils are mainly composed of aromatic and volatile compounds and can be obtained from different parts of plants, especially the leaves and flowers^1^. Indeed, in plants, they are either secreted directly from the protoplasm by the degradation of cell membrane and resin materials or by the hydrolysis of some glycosides^2^. In particular, glycosides produced by different species of plants and stored in different organs have a strict relationship with biosynthesis, metabolism and biological activity and are mostly affected by the environmental climatic conditions^3^. Essential oils are usually rich in terpenes, sesquiterpenes, esters, aldehydes, phenols, ethers and peroxides^3,4^; they are mostly colorless or yellowish, less dense than water and soluble in organic solvents^5^. They are widely used in various industries, including the food and cosmetic industries among others^6^. Moreover, since ancient times, they have been widely used for the treatment of different disorders thanks to their well-known antioxidant, antimicrobial and antifungal properties^7–9^. Currently, essential oils have been proposed and tested in alternative medicine, especially as antimicrobial and antifungal products. Indeed, there is increased demand for safe and effective plant-derived bioactives as an alternative to antimicrobial synthetic drugs because their widespread and continuous use has led to the modification of microbes that have become resistant, thus reducing the therapeutic effect of these drugs^10^. Essential oils derived from plants have demonstrated promising antimicrobial therapeutic effects, which are generally accompanied by reduced side effects^11^. They have been screened and used in pharmacology, herbal pharmacology, medical microbiology and phytopathology^12^ especially because of their known insecticidal, antifungal, anti-parasitic, antibacterial, antiviral, antioxidant and cytotoxic properties^13^. These activities are related to the lipophilic nature of the hydrocarbon skeleton and of the functional groups of the bioactives. Indeed, the hydrophobic properties of the bioactives facilitate their interaction with bacteria and entrance into the cell, where they can exert their therapeutic effect. Rod-shaped cells and Gram-positive bacteria seem to be more sensitive than Gram-negative bacteria to these bioactives^5,14^.

Lamiaceae Martinov is one of the largest plant families in the world (~ 252 genera and 6700 taxa) and has the main differentiation center in the Mediterranean and Irano-Turanian biogeographic regions^15–18^. The majority of Lamiaceae produce terpenes and a wide variety of other compounds, which are mainly stored in the epidermal glands of leaves, stems and reproductive organs^19^.

One of the most important genera of Lamiaceae is *Salvia* L. with about 900 species worldwide and more than 70 species in Iran, 17 of which are endemic and exclusive to Iran^20,21^. Salvia, the name of which is derived from the word salwar which means healer, has been traditionally used as an anti-toxin and a restorative, aimed at strengthening the health and extending the longevity of both humans and soul^22^. The essential oils of *Salvia* species contain various bioactives such as terpenoids, steroids, flavonoids and polyphenols among others^23^ and their concentration varies as a function of the part of the plant used^24^. Indeed, many *Salvia* species and their essential oils are commonly used in pharmaceutical and cosmetic products or used as additives for foods (seasonings and flavors)^25,26^.

Previous studies reported that the main components contained in the essential oils from *S. hydrangea* DC. ex Benth. depend on the part of the plant used and the collection zone. Caryophyllene oxide and β-caryophyllene^10,27^; and β-caryophyllene, 1,8-cineole, α-pinene and caryophyllene oxide^28^ have been identified by different authors. Naphthalene, 1,8-cineole, camphor and α-terpineol are the main bioactives contained in plants at 2000 m above sea level, while 1,8-cineole, camphor, β-pinene, naphthalene and α-amorphene are those contained in plants at 1100 m above sea level^29^. Camphor, α-humulene^30^, α-pinene, 1,8-cineole, trans-caryophyllene and camphene^31^ have been found in the essential oil from aerial parts of *S. hydrangea* and 1,8-cineole, caryophyllene oxide, α-pinene and β-pinene^32^ are the major compounds detected in oil obtained only from the leaves of this plant.

The oil obtained from *S. hydrangea* flowers shows an in vitro anti-malarial effect due to the presence of high levels of pentacyclic triterpenes (mainly oleanic acid) that inhibit the growth of the malaria pathogen^33^. The essential oil from the aerial parts of *S. hydrangea* is effective against different bacteria^10,28,30^.

The present study aimed to investigate essential oil from both leaves and flowers of Iranian *S. hydrangea*. To this purpose, the chemical composition of oils has been determined and compared. Moreover, the variations in yield and antimicrobial activity as a function of the composition have been evaluated.

## Materials and methods

### Plant material

To select the sampling region, at first, habitats of the plant were identified through field surveys. Then, Daran region, located in Isfahan, Iran was selected (longitude: E 46° 49ʹ 02ʺ; latitude: N 36° 54ʹ 170ʺ). To sample the studied plant, in June 2018, coinciding with flowering, three points were selected randomly from Daran region. At each point, leaves and flowers of *S. hydrangea* were collected randomly from different plants (100 plants at each point). The specimens were transferred to the laboratory after being harvested and then exposed to free air to dry. One sample of the whole plant was also collected and pressed. The specimens were identified and recorded in the herbarium of the University of Kashan.

### Isolation of essential oils

After complete drying, the samples were ground using a small electric mill. Each dried plant was weighed (100 g) and subjected to the extraction process by means of water distillation using Clevenger apparatus (made in Germany) for 5 h. The essential oil was dried by anhydrous sodium sulfate and after filtration was stored in dark bottles at 4 °C until use for further studies. Essential oil yield was calculated based on weight percent (w/w). This process was repeated three times for the oil from each plant part.

### Gas chromatography/mass spectrometry (GC–MS) analyses

The main bioactives contained in the essential oils were determined by means of GC–MS, using an Agilent 6890 chromatograph coupled with an N-5973 mass spectrometer. A capillary column (HP-5MS) with a 5% methylphenylsiloxane static phase (length 30 m, internal diameter 0.25 mm, static layer thickness 0.25 μm) and ionization energy of 70 eV was used. The temperature for the analyses was first set at 60 °C and then increased at a rate of 3 °C/min up to 246 °C. The injector and detector temperatures were maintained at 250 °C, the volume of the injected sample was 1 µL and the helium carrier gas was maintained at a flow rate of 1.5 mL/min. Identification of chemical components was based on analysis of the chromatograms obtained for each oil, by means of evaluating the retention indices (RI) in comparison with standards of n-alkane mixtures (C8–C20) and the mass spectral data of each peak using a computer library (Wiley-14 and NIST-14 Mass Spectral Library), and comparison of the results with those contained in the literature^34^.

### Bacterial strains tested

Twelve microorganisms, provided by the Iranian Research Organization for Science and Technology (IROST), were used to evaluate the antimicrobial activity of the essential oils: three Gram-positive bacteria, *Staphylococcus epidermidis* (ATCC 12228), *Staphylococcus aureus* (ATCC 29737) and *Bacillus subtilis* (ATCC 6633), and six Gram-negative bacteria, *Klebsiella pneumoniae* (ATCC 10031), *Shigella dysenteriae* (PTCC 1188), *Pseudomonas aeruginosa* (ATCC 27853), *Salmonella paratyphi-A serotype* (ATCC 5702), *Proteus vulgaris* (PTCC 1182) and *Escherichia coli* (ATCC 10536). Fungal strains were used as well: *Aspergillus niger* (ATCC 16404), *Aspergillus brasiliensis* (PTCC 5011) and *Candida albicans* (ATCC 10231). Bacterial strains were cultured overnight at 37 °C in nutrient agar and fungi were cultured overnight at 30 °C in Sabouraud dextrose agar.

### Agar diffusion method

This procedure was performed according to CLSI standards: 6.0 mm diameter well plates containing Müller Hinton agar were prepared and 100 µL of bacterial suspension with a half-McFarland turbidity equivalent in culture medium were cultured. The essential oils were dissolved in dimethyl-sulfoxide (DMSO) at a concentration of 30 mg/mL; 10 μL (equivalent to 300 μg) of each oil was poured into the wells. The plates were incubated at 37 °C for 24 h for bacterial strains and 48 h and 72 h at 30 °C for yeast and fungi, respectively, and antimicrobial activity was evaluated for each microorganism by measuring the diameter of the inhibition halo (in millimeters), according to an antibiogram ruler. To evaluate the repeatability of the results, three replicates were performed for each essential oil and each strain. DMSO was used as a negative control. Gentamicin (10 µg/disk) and rifampin (5 µg/disk) for bacteria and nystatin (100 I.U.) for yeast were used as standard drugs for positive control in the same conditions as tested oils.

### Minimum inhibitory concentration (MIC)

The minimum concentration able to inhibit the growth of bacteria and yeast was calculated by means of a microdilution method and for fungal strains was calculated by agar dilution assay. Essential oils (2000 μg/mL) were dissolved in a mixture of tryptic soy broth medium and DMSO and then opportunely diluted, using the same mixture, to reach different concentrations (1000, 500, 250, 125, 62.5, 31.25, and 15.63 mg/mL).

Sterile 96-well microplates were filled with 95 µL of culture medium, 5 µL of bacterial suspension with 0.5 McFarland dilution and 100 µL of the essential oil at different concentrations. Then, plates were incubated at 37 °C for 24 h for bacterial strains and 48 h at 30 °C for yeast. The MIC was determined by means of the improvement of opacity or the change in color. The MIC was the lowest concentration of an antimicrobial that inhibited visible growth (absence of turbidity).

### Minimum bactericidal concentration (MBC)

To determine the minimum concentration able to kill the bacteria, the same microdilution method described above was used. After 24 h of incubation with both bacteria and oils at different concentrations, 5 µL of the content of each well was inoculated with nutrient agar medium and incubated at 37 °C for 24 h for bacterial strains. After incubation, the colony-forming units were enumerated. The MBC was the lowest concentration able to effectively reduce the growth of microorganisms (99.5%).

### Statistical analysis

Statistical analysis was performed using SPSS software. First, the normality of the statistical variables was investigated using a Kolmogorov–Smirnov test. After that, to ensure the normality of the data, the variance was analyzed using one-way analysis of variance (ANOVA). Comparison of the means was performed using a Duncan test with a probability level of 5% error.

## Results and discussion

### Chemical composition of essential oils

The leaves or flowers of *S. hydrangea* were separately distilled by steam, resulting in two light yellow essential oils. The yield from leaves was ~ 0.25% and that from flowers was ~ 0.28%, lower than those obtained in previous studies based on extraction of the aerial parts of this plant, but it can be connected to both the species used and climatic growth conditions^28–31^. Indeed, secondary metabolites are generally synthetized in plants as they represent the most important defense mechanisms against pathogens; the amount produced along with the quality may vary as a function of habitat, the organ in which they are produced and climate conditions^35,36^. These differences are most likely due to differences in chemotype, which are also connected to environmental and climate conditions^37^. GC/MS analyses showed that the chemical composition of essential oils was similar even though oil from leaves contained 39 components and that from flowers only 27. They represent 99.96% and 97.85% of all the compounds, respectively (Table 1). The results obtained for flower oil are in agreement with previous studies in which the number of compounds in the aerial parts of this plant varied from 33 to 54^28,30,31^. Differently, Ghannadi et al.^32^ identified 13 compounds in the essential oil obtained from the leaves of this plant species, which represent a small part of those detected in this study. As previously underlined, this difference may be due to growth, genetic and environmental factors^38^.

**Table 1:** Bioactives contained in essential oils from leaves and flowers of *S. hydrangea*.

No.	Compound (%)	RI^a^	Relative percentage		Molecular formula
			Leaf	Flower	
1	α-Thujene	863.546	0.45 ± 0.00^u^	–	C_10_H_16_
2	α-Pinene	871.921	3.61 ± 0.00^h^	1.78 ± 0.01^m^	C_10_H_16_
3	Camphene	888.669	2.53 ± 0.00^j^	0.83 ± 0.00^q^	C_10_H_16_
4	β-Pinene	914.238	8.91 ± 0.00^d^	4.98 ± 0.00^d^	C_10_H_16_
5	β-Myrcene	920.198	0.62 ± 0.01^t^	–	C_10_H_16_
6	α-Terpinene	942.715	0.31 ± 0.00^wx^	–	C_10_H_16_
7	1,8-Cineole	957.615	13.96 ± 0.00^b^	9.54 ± 0.00^b^	C_10_H_18_O
8	1,3,6-Octatriene	966.556	0.42 ± 0.02^uv^	–	C_8_H_12_
9	γ-Terpinene	977.152	0.67 ± 0.00^t^	0.52 ± 0.00^t^	C_10_H_16_
10	α-Terpinolene	1001.587	0.27 ± 0.00^x^	1.28 ± 0.00^o^	C_10_H_16_
11	Linalool	1013.756	2.24 ± 0.06^k^	1.70 ± 0.02^m^	C_10_H_18_O
12	Camphor	1046.031	4.06 ± 0.00f	3.33 ± 0.00^g^	C_10_H_16_O
13	Borneol	1065.343	3.89 ± 0.00^g^	0.96 ± 0.02^p^	C_10_H_18_O
14	α-Terpinen-4-ol	1070.105	0.95 ± 0.01^qr^	0.85 ± 0.02^q^	C_10_H_18_O
15	α-Terpineol	1081.481	1.12 ± 0.02^o^	–	C_10_H_18_O
16	(−)-Bornyl acetate	1136.298	1.76 ± 0.01^m^	1.90 ± 0.02^l^	C_12_H_20_O_2_
17	Thymol	1157.932	1.59 ± 0.01^n^	–	C_10_H_14_O
18	β-Bourbonene	1198.317	3.20 ± 0.07^i^	2.05 ± 0.00^j^	C_15_H_24_
19	cis-Jasmone	1211.611	0.68 ± 0.01^t^	–	C_11_H_16_O
20	trans-Caryophyllene	1221.563	9.58 ± 0.00^c^	6.36 ± 0.00^c^	C_15_H_24_
21	β-Cubebene	1224.881	0.95 ± 0.01^qr^	–	C_15_H_24_
22	γ-Cadinene	1233.175	0.53 ± 0.01^u^	–	C_15_H_24_
23	β-Farnesene	1236.255	1.02 ± 0.00^pq^	0.89 ± 0.03^q^	C_15_H_24_
24	α-Humulene	1239.810	0.72 ± 0.01^t^	0.59 ± 0.00^s^	C_15_H_24_
25	α-Amorphene	1253.791	1.18 ± 0.00^o^	–	C_15_H_24_
26	β-Selinene	1258.767	0.37 ± 0.00^vw^	–	C_15_H_24_
27	β-Bisabolene	1268.009	0.80 ± 0.00^s^	0.59 ± 0.01^s^	C_15_H_24_
28	δ-Cadinene	1277.488	0.63 ± 0.01^t^	–	C_15_H_24_
29	Caryophyllene oxide	1297.156	0.46 ± 0.00^u^	35.47 ± 0.00^a^	C_15_H_24_O
30	(+) Spathulenol	1318.886	16.07 ± 0.00^a^	–	C_15_H_24_O
31	Calarene	1323.170	0.83 ± 0.00^rs^	–	C_15_H_24_
32	(−)-Humulene epoxide II	1331.234	1.06 ± 0.04^op^	1.97 ± 0.00^k^	C_15_H_24_O
33	Widdrene	1343.825	1.45 ± 0.07^n^	–	C_15_H_24_
34	Adamantane	1348.184	–	2.22 ± 0.00^i^	C_10_H_16_
35	Isoaromadendrene epoxide	1348.910	1.82 ± 0.02^m^	–	C_15_H_24_O
36	β-Eudesmol	1357.584	5.33 ± 0.00^e^	4.11 ± 0.00^e^	C_15_H_26_O
37	Valencene	1359.806	–	1.39 ± 0.02^n^	C_15_H_24_
38	Valeranone	1365.859	1.81 ± 0.06^m^	3.32 ± 0.02^g^	C_15_H_24_O
39	Caryophyllenol-II	1368.523	2.03 ± 0.01^l^	3.46 ± 0.00f	C_15_H_24_O
40	Calamenene	1375.786	0.68 ± 0.00^t^	–	C_15_H_22_
41	Phthalic acid	1462.468	–	0.70 ± 0.04^r^	C_8_H_6_O4
42	Palmitic acid	1515.526	0.90 ± 0.02^r^	2.98 ± 0.00^h^	C_16_H_32_O_2_
43	p-Cymene	1583.157	–	2.06 ± 0.01^j^	C_10_H_14_
44	trans-Oleic acid	1600.831	–	2.02 ± 0.02^j^	C_18_H_34_O_2_
	Total		99.96	97.85	
	Monoterpenes hydrocarbons		17.37	13.65	
	Oxygenated monoterpenes		27.81	16.38	
	Sesquiterpenes hydrocarbons		21.94	11.87	
	Oxygenated sesquiterpenes		28.58	48.33	
	Others		3.76	7.6	

^a^Retention indices (RIs) relative to n-alkanes (C6–C40) on the same methyl silicone capillary column. Values with different letters are statistically different (Duncan, p ≤ 0.05).

The most representative volatile constituents of *S. hydrangea* essential oils were oxygenated sesquiterpenes (28.58% in leaf oil and 48.33% in flower oil), in accordance with previous results^39^. Oxygenated monoterpenes were also present but in lower amounts (27.81% in leaf oil and 16.38% in flower oil), but these results were not in agreement with those obtained by Kotan et al.^30^, who detected a higher amount of these compounds. These differences may be related to the timing of plant collection and the ecological conditions^38^.

**Figure 1:**
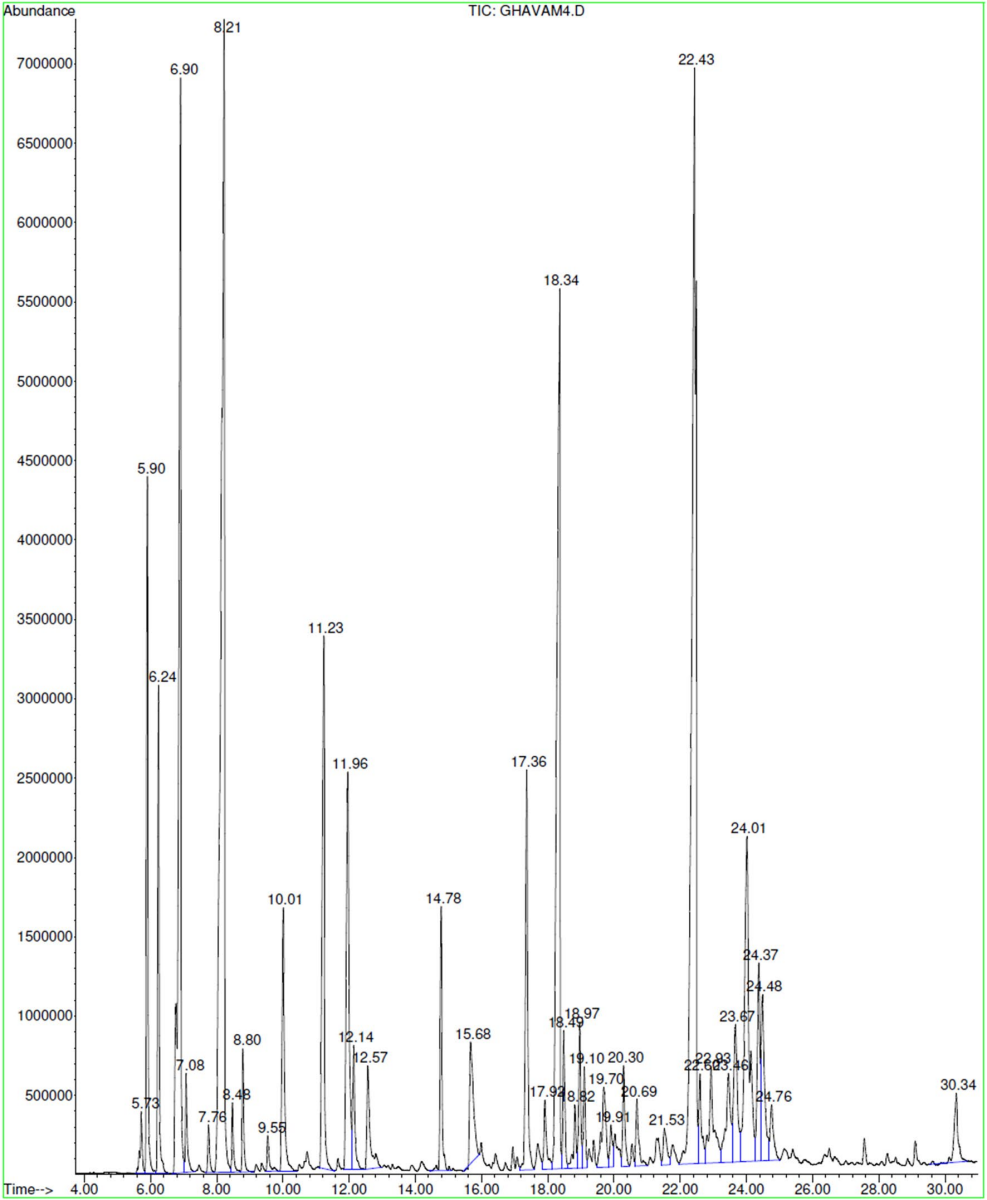
Representative GC–MS chromatogram of essential oil from leaves of *S. hydrangea*.

The ANOVA results showed that there was a significant difference between the mean of the components obtained for each of the essential oils of *S. hydrangea* flowers and leaves (P ≤ 0.05). The most abundant components in the essential oil from leaves were (+)-spathulenol (16.07%), 1,8-cineole (13.96%), trans-caryophyllene (9.58%), β-pinene (8.91%), β-eudesmol (5.33%), camphor (4.06%) and α-pinene (3.61%) (Table 1 and Fig. 1), in accordance with the findings of Ghannadi et al.^32^. However, (+)-spathulenol, β-eudesmol, trans-caryophyllene and camphor were detected for the first time in this study. Again, habitat and climate changes may affect the composition of the extractive products, as growth and growth stages, farming and genetic characteristics may vary significantly^40^.

The most abundant component in the essential oil from leaves was spathulenol, an alcoholic sesquiterpene with a primary skeleton similar to that of azulene. It has antibacterial and antifungal properties along with anti-inflammatory and anti-cancer activity and is also considered an inducer of apoptosis^41^. Spathulenol can be also used as a pesticide.

**Figure 2:**
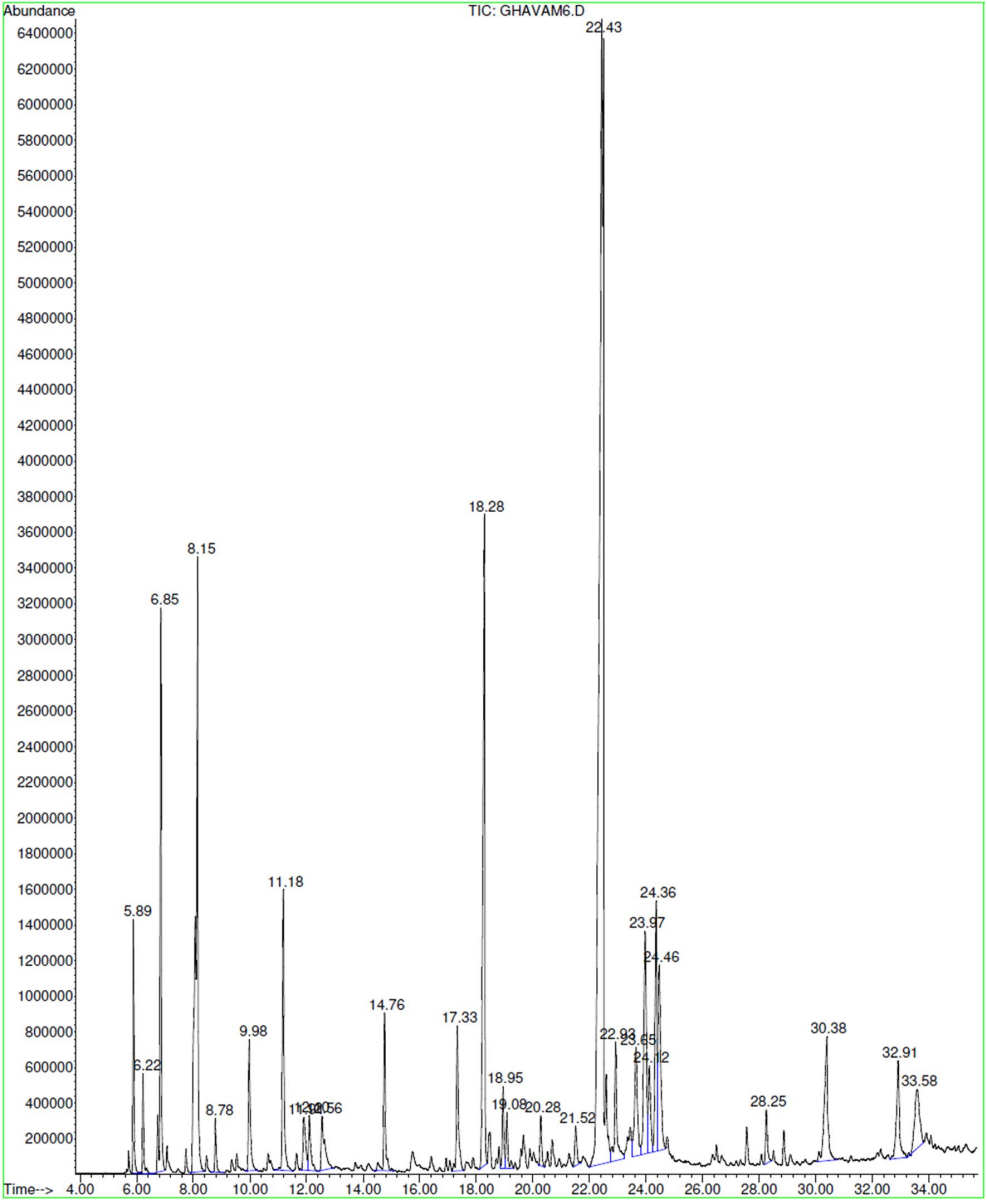
Representative GC–MS chromatogram of essential oil from flowers of *S. hydrangea*.

Caryophyllene oxide (35.47%), 1,8-cineole (9.54%), trans-caryophyllene (6.36%), β-eudesmol (4.11%), caryophyllenol-II (3.46%) and camphor (3.33%) were the most abundant compounds in the oil from *S. hydrangea* flowers (Table 1; Fig. 2). Caryophyllene oxide (55.4%) was also the most abundant component detected from the epigean parts of this plant in a previous study^39^, although other studies showed different results, as the amount of this component varied from 25.4^27^ to 8.6%^28^. Caryophyllene oxide inhibits abnormal fluid accumulation in the intercellular spaces of both healthy and tumor tissues^42^.

1,8-Cineole (9.54%) was the second most abundant bioactive in this essential oil, as reported previously by other authors, with some differences in the amount detected: 18.08%^31^, 15.2%^28^ and 9.45% (at elevations of 1100 m above sea level^29^). 1,8-Cineole has been successfully used in pharmaceutical and cosmetic fields thanks to its anti-parasitic and antifungal activity and insect-repellent properties. Moreover, it has been used as a key component in topical mouthwashes thanks to its analgesic properties^43,44^.

Trans-caryophyllene was detected in a high amount as well (6.36%) and it was the third main component of flower oil. This result is in agreement with that obtained previously by Mahdiyan et al.^31^, who detected a higher amount (17.38%) of this bioactive as it was also the third most abundant in their extractive products.

The amount of β-eudesmol detected (4.11%) was slightly lower than that found in the essential oil previously prepared (5.22%) by Ebrahimi et al.^29^. This small difference may be connected to the different habitat and altitude in which the same plant was grown. β-Eudesmol has been used in traditional medicine mainly because of its diuretic, anti-hypertensive, antipyretic, antiseptic and antimicrobial properties^45–47^.

Caryophyllene-II was detected for the first time in this study in the essential oil obtained from the flowers. A small amount of camphor was detected. Differently, it was the second main bioactive detected in the essential oil obtained by Ebrahimi et al.^29^; in particular, the amount was higher (12.06%) when the plant was cultivated at 1100 m above sea level and lower (5.71%) when cultivated at 2000 m above sea level. These differences confirm the key role of the environment (soil chemical composition and physiographic factors such as altitude) on the genetic and non-genetic variations of plants along with the production of secondary metabolites^35,36,48^. Camphor is a monoterpene, widely used in traditional and modern medicine thanks to its antimicrobial properties and beneficial effects on the cardiovascular system. Moreover, it has been used as a topical anti-itching treatment, insect repellent, anti-inflammatory and analgesic^49,50^. It is well accepted by patients as it has a bitter taste. In addition, its low solubility in aqueous solvents makes it an excellent candidate to be delivered in nanocarriers^51^.

### Antimicrobial activity

Essential oils are traditionally used as antibacterial and antifungal agents in natural medicine. The increasing interest of modern society and the pharmaceutical industry for medicinal plants, makes crucial the scientific studies aimed at confirming these effects and founding new therapeutic agents^52^.

In this study, considering their promising composition, the antibacterial and antifungal activity of essential oils from *S. hydrangea* leaves or flowers was assayed (Table 2). The ANOVA results showed that there was a significant difference between the mean inhibition halos obtained on treating different microorganisms with the essential oil of flowers and leaves of *S. hydrangea* and antibiotics (P ≤ 0.05). The essential oil from *S. hydrangea* leaves was especially active against Gram-positive bacteria including *Bacillus subtilis*, *Staphylococcus epidermidis* and *Staphylococcus aureus* as the inhibition halo was large irrespective of the concentration used (~ 9.50 mm), even if it was significantly lower than that obtained with rifampin (19, 44 and 21 mm) and gentamicin (30, 39 and 27 mm). Similarly, Kotan et al.^30^ reported a weak antibacterial effect of the essential oil against *Staphylococcus aureus* (8 mm inhibition halo), while Sonboli et al.^28^ and Asadollahi et al.^39^, detected a better antibacterial activity, especially against *Bacillus subtilis* and *Staphylococcus epidermidis* which showed inhibition halos of 17 and 16 mm, respectively.

**Table 2:** Inhibition halo (IH) diameter, minimum inhibitory concentration (MIC) and minimum bactericidal concentration (MBC) obtained on treating microorganisms with the essential oils from leaves and flowers of *S. hydrangea*.

Microorganism	Leaves			Flowers			Antibiotics					
							Rifampin		Gentamicin		Nystatin	
	IH (mm)	MIC (µg/mL)	MBC (µg/mL)	IH (mm)	MIC (µg/mL)	MBC (µg/mL)	IH (mm)	MIC (µg/mL)	IH (mm)	MIC (µg/mL)	IH (mm)	MIC (µg/mL)
*Shigella dysenteriae*	ND	62.5	62.5	ND	62.5	125	9 ± 0.00^b^	15.63	17 ± 0.01^a^	3.90	NA	NA
*Pseudomonas aeruginosa*	ND	15.63 >	15.63 >	ND	15.63 >	15.63 >	ND	31.25	20 ± 0.00^a^	7.81	NA	NA
*Bacillus subtilis*	9.50 ± 0.00 ^c^	250	> 1000	ND	31.25	31.25	19 ± 0.00^b^	31.25	30 ± 0.02^a^	3.90	NA	NA
*Staphylococcus epidermidis*	9.50 ± 0.00^d^	1000	1000	10.67 ± 1.15^c^	250	250	44 ± 0.01^a^	1.95	39 ± 0.00^b^	1.95	NA	NA
*Escherichia coli*	ND	1000	1000	ND	31.25	125	10 ± 0.00^b^	15.63	23 ± 0.02^a^	31.25	NA	NA
*Staphylococcus aureus*	9.50 ± 0.50 ^c^	500	> 1000	ND	125	125	21 ± 0.01^b^	31.25	27 ± 0.03^a^	1.95	NA	NA
*Klebsiella pneumoniae*	ND	62.50	62.5	ND	62.5	62.5	8 ± 0.00^b^	15.63	17 ± 0.03^a^	3.90	NA	NA
*Proteus vulgaris*	ND	250	250	ND	125	125	8 ± 0.00^b^	15.63	24 ± 0.04^a^	15.63	NA	NA
*Salmonella paratyphi-A*	ND	125	125	ND	62.5	125	8 ± 0.01^b^	15.63	18 ± 0.01^a^	3.90	NA	NA
*Candida albicans*	ND	250	250	ND	1000	1000	NA	NA	NA	NA	33 ± 0.01^a^	125
*Aspergillu sniger*	ND	> 2000	> 2000	ND	2000	2000	NA	NA	NA	NA	27 ± 0.00^a^	31.2
*Aspergillus brasiliensis*	ND	> 2000	> 2000	ND	2000	2000	NA	NA	NA	NA	30 ± 0.01^a^	31.2

NA indicates no activity and ND indicates not determined. Mean values ± standard deviations of three cultures were reported (n = 3).

Values with different letters are statistically different (Duncan, p ≤ 0.05).

The essential oil from *S. hydrangea* flowers did not show any inhibition halo, except against the Gram-positive *Staphylococcus epidermidis* (~ 11 mm). It was significantly lower in comparison with those obtained after treatment with rifampin (~ 44 mm) and gentamicin (~ 39 mm).

The antibacterial activity of essential oils, especially that of the essential oil obtained from leaves, seems to be mainly related to the presence of two monoterpenes: α-pinene and β-pinene. The first is widely used in the manufacture of insecticides, sprays and disinfectants. Further, it has anti-inflammatory, antibacterial and anti-cancer properties, along with spasmolytic and skin redness properties. β-Pinene is generally used to manufacture aromatic oil and as a monomer in the production of terpene resins; moreover, it has anti-inflammatory and antibacterial activity. Both monoterpenes (α- and β-pinene) have antimicrobial activity against Gram-positive and Gram-negative bacteria, especially *Escherichia coli*, *Staphylococcus bacteria*^53,54^, *Staphylococcus aureus* and *Bacillus subtilis*^55,56^.

Essential oil from leaves or flowers of *S. hydrangea* was also effective in inhibiting the growth of bacteria. MIC values varied from 15.63 to 2000 µg/mL as a function of the organism tested and the oil used. The lowest MIC was found against *Pseudomonas aeruginosa* (15.63 μg/mL) irrespective of the oil used. This value was half that provided by rifampin (31.25 μg/mL) and double that for gentamicin (7.81 µg/mL). Moreover, these results are encouraging as in previous studies any inhibitory effect was detected by using essential oil from the aerial part of the same plant^28,39^.

The highest MIC (2000 µg/mL) was detected for *Aspergillus brasiliensis* and *Aspergillus niger*, which showed a high resistance to both oils. Inhibition of the growth of Gram-negative bacteria such as *Staphylococcus epidermidis* and *Escherichia coli* was low for leaf oil (MIC = 1000 µg/mL), but always higher than that obtained by Sonboli et al.^28^ and Asadollahi et al.^39^, who found an MIC of 1500 µg/mL against *Escherichia coli*. Surprisingly, the MIC (31.2 µg/mL) of flower oil against *Escherichia coli*, which is the main diarrhea-causing agent in humans, was significantly higher in comparison with the corresponding oil from leaves. It was similar to that provided by gentamicin (31.25 µg/mL), but significantly lower than that for rifampin (15.63 µg/mL).

The MIC value of *S. hydrangea* essential oils was the same against the Gram-negative bacteria *Shigella dysenteriae* (62.5 µg/mL), *Pseudomonas aeruginosa* (15.63 µg/mL) and *Klebsiella pneumoniae* (62.5 µg/mL), probably because of the similar composition of the two oils. However, the differences detected in the composition of the oils may be responsible for the greater inhibitory effect of the essential oil obtained from flowers against Gram-positive bacteria like *Bacillus subtilis*, *Staphylococcus epidermidis* and *Staphylococcus aureus* and Gram-negative bacteria such as *Escherichia coli*, *Proteus vulgaris* and *Salmonella paratyphi-A*. On the contrary, efficacy against *Candida albicans* was higher when the oil from leaves was used (MIC 250 µg/mL versus 1000 µg/mL provided by flower oil), especially when compared to the commercial antifungal nystatin (125 µg/mL).

In general, the ability of these essential oils to inhibit the growth of different bacterial strains was lower than that of both rifampin and gentamicin used as controls.

Essential oils from both leaves and flowers showed very low or no effectiveness against Gram-negative bacteria, in agreement with previous results^28^.

Other studies have shown that Gram-negative bacteria are more resistant to plant-derived essential oils in comparison with Gram-positive bacteria, probably because of the different composition of the lipopolysaccharide membrane. Indeed, the cell wall structure of Gram-negative bacteria is more complex than that of Gram-positive bacteria^57,58^. Due to the similar composition of oils from leaves and flowers, as reported above, the antibacterial activity can be attributed to the monoterpenes α- and β-pinene. However, since the amount of these two compounds was lower in the oil from flowers (Table 1), the inhibition of *Staphylococcus epidermidis* was also lower.

The MBC value of essential oils from both leaves and flowers varied from 15.63 to 2000 µg/mL. The results underline that the MBC values provided by the oil from leaves were always equal to the MIC values except for that for *Bacillus subtilis*. Differently, MBC values provided by flower oil were higher than MIC values against Gram-negative bacteria (*Shigella dysenteriae*, *Escherichia coli*, *Proteus vulgaris* and *Salmonella paratyphi-A*), while they were similar for all the other microorganisms treated. To the best of our knowledge, the MBC has been investigated for the first time in this work, thus no comparison can be made with previous studies.

The essential oils from *S. hydrangea* leaves and flowers have a significant inhibitory and lethal effect, especially against the Gram-negative bacterium *Pseudomonas aeruginosa* for which the lowest MIC and MBC values were found (15.63 µg/mL). This bacterium may cause opportunistic and often nosocomial infections. This efficacy may be specific to the essential oils from the leaves and flowers of *S. hydrangea*. Indeed, the activity of the genus *Salvia* against *Pseudomonas aeruginosa* is strictly dependent on the species used. Many species of the genus *Salvia* do not show any activity against this bacterium and a significant effect has only been reported for *Salvia mirzayanii* Rech.f. & Esfand.^59^. Similarly, the antimicrobial effect against the bacteria *Shigella dysenteriae* and *Klebsiella pneumoniae*, which may cause diarrhea and pneumonia, may vary as a function of the species used. In general, all *Salvia* species have significant antibacterial activity against both Gram-positive and Gram-negative bacteria (*Bacillus*, *Klebsiella*, *Pseudomonas* and *Salmonella*) and yeasts (*Candida* and *Aspergillus*). Therefore, the antimicrobial properties of *Salvia* against food spoilage and food-borne pathogens suggest its use as a natural preservative in food applications^60,61^.

As reported above, the antibacterial activity of essential oils obtained from different species of *Salvia* is mainly connected to their composition, especially oxygenated monoterpenes, which are present in high amounts^62,63^. They act by destroying the microbial cytoplasmic wall, improving its permeability and allowing the passage of large protons and ions. However, it is difficult to attribute the antibacterial effect to a specific compound because the essential oils obtained for different species may contain different mixtures of bioactives^64^. Further, due to the complexity of the composition of the essential oils, it is also difficult to explain the mechanism of action of these blends, but is important to underline that the wide variety of composition is a positive factor that may limit the development of resistance which is otherwise very common for synthetic drugs. In light of this, essential oils may represent a valid alternative to avoid the multidrug resistance of many pathogens, or they could be used in combination with antimicrobials to improve their effectiveness against different infectious diseases^65^. In addition, several studies have suggested that their delivery into ad hoc formulated carriers may improve their efficacy^66^.

## Conclusions

The main component of the two essential oils obtained from leaves and flowers of *S. hydrangea* were spathulenol, 1,8-cineole, trans-caryophyllene, β-pinene, β-eudesmol, camphor, α-pinene and caryophyllene oxide. Both oils had significant inhibitory and lethal effects on the Gram-negative bacterium *Pseudomonas aeruginosa*, that is not common among different *Salvia* species. In addition, both were able to inhibit *Shigella dysenteriae* and *Klebsiella pneumoniae*, which are responsible for diarrhea and pneumonia in humans. Overall, the results underline the potential application of these oils for future development of new therapeutic systems and drug adjuvants, while additional tests should be performed to evaluate their effectiveness as food preservatives. This is a very interesting prospect, which has gained large interest in the last two decades due to awareness concerning the toxicity, ineffectiveness, antibiotic resistance and adverse effects provided by the widespread use of synthetic drugs and food preservatives.

## References

[1] Sharifi-Rad J (2017). Biological activities of essential oils: From plant chemoecology to traditional healing systems. Molecules.

[2] Rao V (2012). Phytochemicals A Global Perspective of Their Role in Nutrition and Health.

[3] Omidbeigy R (1995). Approaches to the Production and Processing of Medicinal Plants Vol II.

[4] Miguel MG (2010). Antioxidant and anti-inflammatory activities of essential oils: A short review. Molecules.

[5] Nazzaro F, Fratianni F, De Martino L, Coppola R, De Feo V (2013). Effect of essential oils on pathogenic bacteria. Pharmaceuticals.

[6] Burt S (2004). Essential oils: Their antibacterial properties and potential applications in foods—a review. Int. J. Food Microbiol..

[7] -Buchbauer, G., Jäger, W., Jirovetz, L., Ilmberger, J. & Dietrich, H. *Therapeutic Properties of Essential Oils and Fragrances *Vol 525, 159–165 (ACS Symposium Series, 1993).

[8] Dagli N, Dagli R, Mahmoud RS, Baroudi K (2015). Essential oils, their therapeutic properties, and implication in dentistry: A review. J. Int. Soc. Prev. Community Dent..

[9] Edris AE (2007). Pharmaceutical and therapeutic Potentials of essential oils and their individual volatile constituents: A review. Phyther. Res..

[10] Abdollahi A, Koohpayeh SA, Najafipoor S, Mansoori Y, Abdollahi S (2013). Evaluation of drug resistance and Staphylococcal cassette chromosome (SCCmec) types among methicillin-resistant *Staphylococcus aureus* (MRSA). J. Alborz Health.

[11] Meshkibaf MH, Abdollahi A, Fsihi Ramandi M, Adnani Sadati SJ, Moravvej A (2011). Antibacterial effects of hydro-alcoholic extracts of *Ziziphora tenuior*, *Teucrium polium*, Berberis corcorde and *Stachys inflata*. Koomesh.

[12] Defera DJ, Ziogas BN, Polission MG (2000). GC/MS analysis of essential oils from some Greek aromatic plants and their fungitoxity on *Penicillum digitatum*. J. Agric. Food Chem..

[13] Kordali S, Kotan R, Mavi A, Cakir A, Ala A, Yildirim A (2005). Determination of the chemical composition and antioxidant activity of the EO of *Artemisia dracunculus* and the antifungal and antibacterial activities of *Artemisia absinthium* and *Artemisia spicigera* essential oils. J. Agric. Food Chem..

[14] Khorshidian N, Yousefi M, Khanniri E, Mortazavian AM (2018). Potential application of essential oils as antimicrobial preservatives in cheese. Innov. Food Sci. Merg. Technol..

[15] Cantino PD, Harley RM, Wagstaff SJ, Harley RM, Reynolds T (1992). Genera of Labiatae: Status and classification. Advances in Labiate Science.

[16] Cantino PD, Olmstead RG, Wagstaff SJ (1997). A comparison of phylogenetic nomenclature with the current system: A botanical case study. Syst. Biol..

[17] Kadereit JW (2004). The Families and Genera of Vascular Plants Vol 7.

[18] Bendiksby M, Thorbek L, Scheen AC, Lindqvist C, Ryding O (2011). An updated phylogeny and classification of Lamiaceae subfamily Lamioideae. Taxon.

[19] Baghalian K, Naghdibadi H (2000). Essential Plants.

[20] Hedge IC (1960). Notes on some cultivated species of *Salvia*. J. R. Hortic. Soc..

[21] Mozaffarian V (1996). A Dictionary of Iranian Plant Names.

[22] Zargari A (2012). Medicinal Plants.

[23] Shirota O, Nagamatsu K, Sekita S (2006). Neoclerodane diterpenes from the hallucinogenic Sage *Salvia divinorum*. J. Nat. Prod..

[24] Lakušić BS, Ristić MS, Slavkovska VN, Stojanović DL, Lakušić DV (2013). Variations in EO yields and compositions of *Salvia officinalis* (Lamiaceae) at different developmental stages. Bot. Serbica.

[25] Lambert Ortiz E (1996). Encyclopedia of Herbs, Spices and Flavouring.

[26] Demirci B, Baser KHC, Yildiz B, Bahcecioglu Z (2003). Composition of essential oils of six endemic *Salvia* spp. from Turkey. Flav. Frag J..

[27] Barazandeh M (2004). Volatile constituents of the oil of *Salvia hydrangea* DC. ex Benth. from Iran. J. Essent. Oil Res..

[28] Sonboli A, Kanani M, Yousefzadi M, Mojarad M (2009). Chemical composition and antibacterial activity of the EO of *Salvia hydrangea* from two localities of Iran. Iran. J. Med. Plants.

[29] Ebrahimi M, Ranjbar S (2016). Essential oils of *Salvia hydrangea* DC. ex Benth. from Kiasar-Hezarjarib regions, Iran-impact of environmental factors as quality determinants. J. Med. Plants. By-prodt..

[30] Kotan R, Kordali S, Cakir A, Kesdek M, Kaya Y, Kilic H (2008). Antimicrobial and insecticidal activities of EO isolated from Turkish *Salvia hydrangea*. Biochem. Syst. Ecol..

[31] Mahdiyan F, Ghasemi Pirbalouti A, Malekpoor F (2016). Qualitative and quantitative changes in the EO of sage (*Salvia hydrangea* DC. Ex Benth.) as affected by different drying methods. J. Herbal Drug.

[32] Ghannadi A, Samsam-Shariat H, Moattar F (1999). Composition of the leaf oil of *Salvia hydrangea*DC. ex Benth. grown in Iran preview access options. J. Essential Oil Res..

[33] Sairafianpour M, Bahreininejad B, Witt M, Ziegler HL, Jaroszewski JW, Staerk D (2003). Terpenoids of *Salvia hydrangea*: Two new, rearranged 20-norabietanes and the effect of oleanolic acid on erythrocyte membrane. Planta Med..

[34] Adams RP (2007). Identification of Essential Oil Components by Gas Chromatography/Quadruple Mass Spectroscopy.

[35] Zargoosh Z, Ghavam M, Bacchetta G, Tavili A (2019). Effects of ecological factors on the antioxidant potential and total phenol content of *Scrophularia striata* Boiss.. Sci. Rep..

[36] Moradi H, Ghavam M, Tavili A (2020). Study of antioxidant activity and some herbal compounds of *Dracocephalum kotschyi* Boiss. in different ages of growth. Biotech. Rep..

[37] Yavari AR, Nazeri V, Sefidkon F, Hassani ME (2010). Evaluation of some ecological factors, morphological traits and EO productivity of *Thymus migricus* Klokov & Desj-Shost. Iran. J. Med. Aro Plants.

[38] Omidbeigy R (2005). Production and Processing of Medicinal Plants Vol 1.

[39] Asadollahi M, Firuzi O, Heidary Jamebozorgi F, Alizadeh M, Jassbi AR (2018). Ethnopharmacological studies, chemical composition, antibacterial and cytotoxic activities of essential oils of eleven *Salvia* in Iran. J. Herbal Med..

[40] Millauskas G, Venskutonis PR, Van Beek TA (2004). Screening of radical scavenging activity of some medicinal and aromatic plant extracts. Food Chem..

[41] Taherkhani M, Masoudi Sh, Fatah Elahi R, Baradari T, Rustayian A (2012). Identify the compounds in the essential oils of two plants from the Apiaceae family, *Reichenb Torilis leptophylla* and Boiss *Thecocarpus meifolius*, and study their antibacterial properties. Iran. J. Chem. Chem. Eng..

[42] Jaimand, K. & Rezaei, MB. *Essential Oil, Distillers, Test Methods and Inhibition Index in EO Analysis*. First Edition. 350 (Publication of the Medicinal Plants Association, 2006).

[43] Francisco JC, Sivik B (2002). Solubility of three monoterpenes, their mixtures and eucalyptus leaf oils in dense carbon dioxide. J. Super Fluid..

[44] Boland DJ, Brophy JJ, House AP (1991). Eucalyptus Leaf Oil Use Chemistry, Distillation and Marketing.

[45] Kusuma IW, Ogawa T, Itoh K, Tachibana S (2004). Isolation and identification of an antifungal sesquiterpene alcohol from Amboyna wood. Pak. J. Biol. Sci..

[46] Ding HY, Wu YC, Lin HC (2000). Phytochemical and pharmacological studies on Chinese changzhu. J. Chin. Chem. Soc..

[47] Marinho CGS, Della Lucia TMC, Guedes RNC, Ribeiro MMR, Lima ER (2005). β-eudesmol-induced aggression in the leaf-cutting ant Atta sexdens rubropilosa. Entomol. Exp. Appl..

[48] Hasani J, NikBaher Z (2014). Evaluation of ecological needs different species of thyme in Kurdistan Habitats. Echo J. Medil Plant..

[49] Majruhi AA (2008). Study of variation in quantity and quality of the EO of *Zhumeria majdae* Rech. F. at different growth stages. J. Med. Plants..

[50] Majdjabari T, Rustaiyan A, Vatanpuor H (2003). Study the ingredients of in essential oil. *Tanacetum khorassanicum* (Krasch). Parsat. J. Med. Plants..

[51] Manconi M (2018). *Thymus* essential oil extraction, characterization and incorporation in phospholipid vesicles for the antioxidant/antibacterial treatment of oral cavity diseases. Colloids Surf. B Biointerfaces.

[52] Baptista-Silva S, Borges S, Ramos OL, Pintado M, Bruno S (2020). The progress of essential oils as potential therapeutic agents: A review. J. Essential Oil Res..

[53] Mohammadi N, Ghasemi A, Aghabarari B, Hamehi B (2016). EOmixtures, anti-bacterial and antioxidant activity of EO of *Nigella sativa* L. different ecotypes in different habitats Iran. Ecol. Chem. J. Med Plants.

[54] Dhar P (2014). Synthesis, antimicrobial evaluation, and structure activity relationship of α-pinene derivatives. J. Agric. Food Chem..

[55] Gilsic S, Milojeij S, Dimitrjvi J, Orlovij A, Skala D (2007). Antimicrobial activity of the EO and different fractions of *Juniperus communis* L. and a comparison with some commercial antibiotics. J. Serb. Chem. Soc..

[56] Leite AM (2007). Inhibitory effect of β-pinene, α-pinene and eugenol on the growth of potential infectious endocarditis causing Gram-positive bacteria. Rev. Bras. Ciên Farma.

[57] Elshafie HS, Ghanney N, Mang SM, Ferchichi A, Camele I (2016). An in Vitro attempt for controlling severe phytopathogens and human pathogens using essential oils from Mediterranean plants of genus *Schinus*. J. Med. Food..

[58] Vikram A, Tripathi DN, Ramarao P, Jena GB (2007). Evaluation of streptozotocin genotoxicity in rats from different ages using the micronucleus assay. Regul. Toxicol. Pharmacol..

[59] Moshefim MH, Mehrabini M, Zolhasb H (2004). Investigating the antimicrobial effects of Iranian sage and Azerbaijani sage extracts on six Gram-positive and Gram-negative microbial strains. J. Kerman Uni. Med. Sci..

[60] Tajkarimi MM, Ibrahim SA, Cliver DO (2010). Antimicrobial herb and spice compounds in food. Food Control.

[61] Sharifi-Rad M (2018). *Salvia* spp. Plants-from farm to food applications and phytopharmacotherapy. Trends Food Sci. Technol..

[62] Norouzi-Arasi H (2005). Volatile constituents and antimicrobial activities of *Salvia suffruticosa* Montbr. & Auch. ex Benth. from Iran. Flavour Fragr. J..

[63] Tepe B, Donmez E, Unlu M (2004). Antimicrobial and antioxidative activities of the essential oils and methanol extracts of *Salvia cryptantha* and *Salvia multiculis*. J. Food Chem..

[64] Lopes-Lutz D, Alviano DS, Alviano CS, Kolodziejcyk PP (2008). The cardiovascular actions of the volatile oil of the black seed (*Nigella sativa*) in rats: Elucidation of the mechanism of action. Gene Pharm..

[65] Salem N (2018). Variation in chemical composition of *Eucalyptus globulus* EO under phonological stages and evidence synergism with antimicrobial standards. Ind. Crops Prod..

[66] Usach I (2020). Comparison between citral and pompia essential oil loaded in phospholipid vesicles for the treatment of skin and mucosal infections. Nanomat (Basel Switzerland).

